# Utilization of scalp cooling to prevent chemotherapy-induced alopecia in woman of skin of color with type 3 hair

**DOI:** 10.1016/j.jdcr.2023.10.018

**Published:** 2023-11-03

**Authors:** Warner Robinson, Beth N. McLellan

**Affiliations:** Division of Dermatology, Department of Medicine, Albert Einstein College of Medicine, Bronx, New York

**Keywords:** alopecia, Black, chemotherapy-induced alopecia, hair loss, scalp cooling, skin of color

## Introduction

Chemotherapy-induced alopecia (CIA) is a frequent side effect of antineoplastic therapy for cancer. It occurs when chemotherapy drugs, such as taxanes, anthracyclines, and alkylating agents, target rapidly dividing cells, damaging the hair follicle.^1^ CIA affects up to 65% of individuals receiving chemotherapy, with up to 8% refusing treatment because of fear of development of CIA.^2^ More than half of female cancer survivors consider CIA to be the most upsetting side effect of antineoplastic therapy.^3^ Women with Afro-textured hair face specific challenges on their cancer journey because for some Black women, hair is an expression of identity—from personal to cultural and even political.^4^

Scalp cooling (SC) has proven to be the most effective preventive therapy for CIA, with some studies reporting up to 75% success rates.^5^ SC works by constricting the blood vessels beneath the skin of the scalp, which reduces the amount of chemotherapy that reaches the hair follicles. SC also decreases the hair follicles’ metabolic activity, which slows cell division and protects the follicles from chemotherapy.^6^ There are few studies regarding the efficacy of SC in patients of African descent and conflicting data about whether SC works on the type 3 (curly) and type 4 (kinky) hair that are characteristic of this racial group. One study suggested that SC was not successful in Black women because of an increase in hair thickness and volume when their hair is prepared with water for SC, minimizing contact between the cooling cap (CC) and scalp and decreasing SC efficacy.^7^ Here, we describe an Afro-Caribbean woman who had success with SC utilizing novel hair preparation techniques developed through the review of available literature and in consultation with SC experts that decreases hair volume and increases contact between the CC and scalp.

## Case description

A 58-year-old postmenopausal Haitian woman with left breast infiltrating ductal cell carcinoma status after lumpectomy with sentinel lymph node biopsy revealing poorly differentiated invasive ductal carcinoma, with 1/1 positive sentinel lymph node (micrometastasis). Estrogen receptor+ (91%-100%); progesterone receptor+ (61%-70%); human epidermal growth factor receptor 2-; and Antigen Kiel 67 (70%). An oncotype showed a recurrence score of 31. Four cycles of adjuvant docetaxel and cyclophosphamide was recommended, and she elected to proceed with SC with the chemotherapy infusions. Her hair preparation was as follows:

Step 1: The hair was parted down the middle hairline and evenly into sections on either side, spreading the hair evenly across the scalp.

Step 2: A thick conditioner was worked through the hair, then water was added with a spray bottle to create an emulsion, allowing the hair to be slicked down to decrease hair volume.

Step 3: The hair was wet thoroughly down to the roots with spray bottle to allow heat to leave the scalp more easily.

Step 4: The hair was then styled in small and loose twists to make the hair as flat as possible.

**Fig 1:**
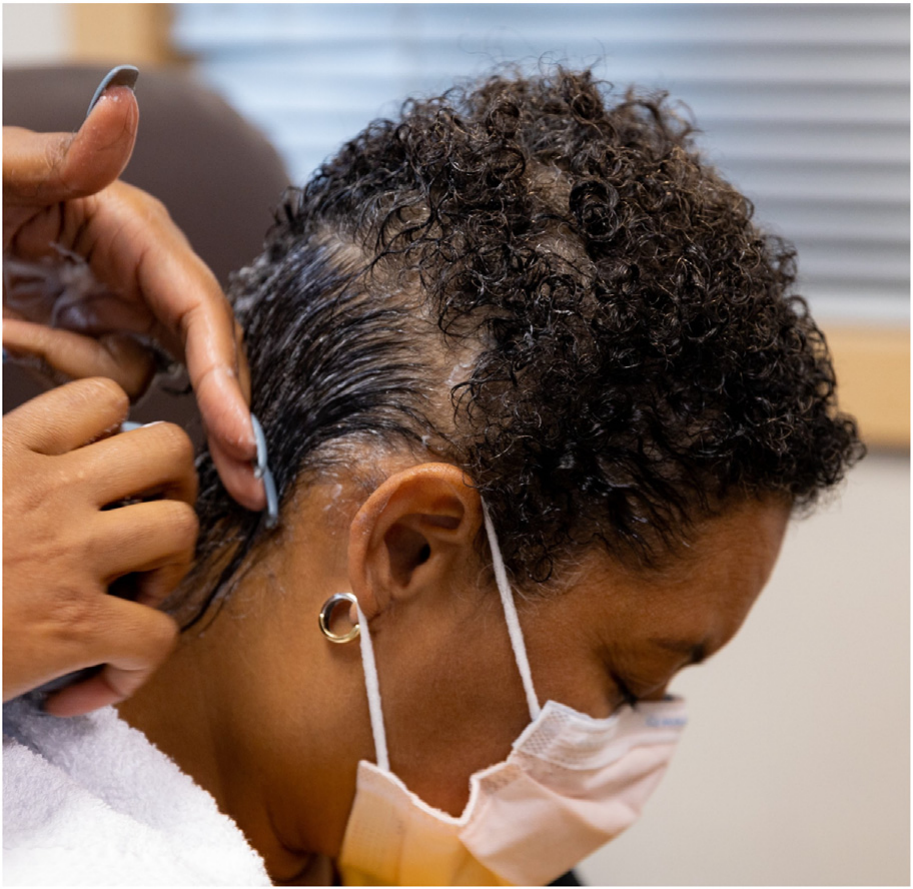
Grade 0 alopecia (no hair loss) at baseline.

**Fig 2:**
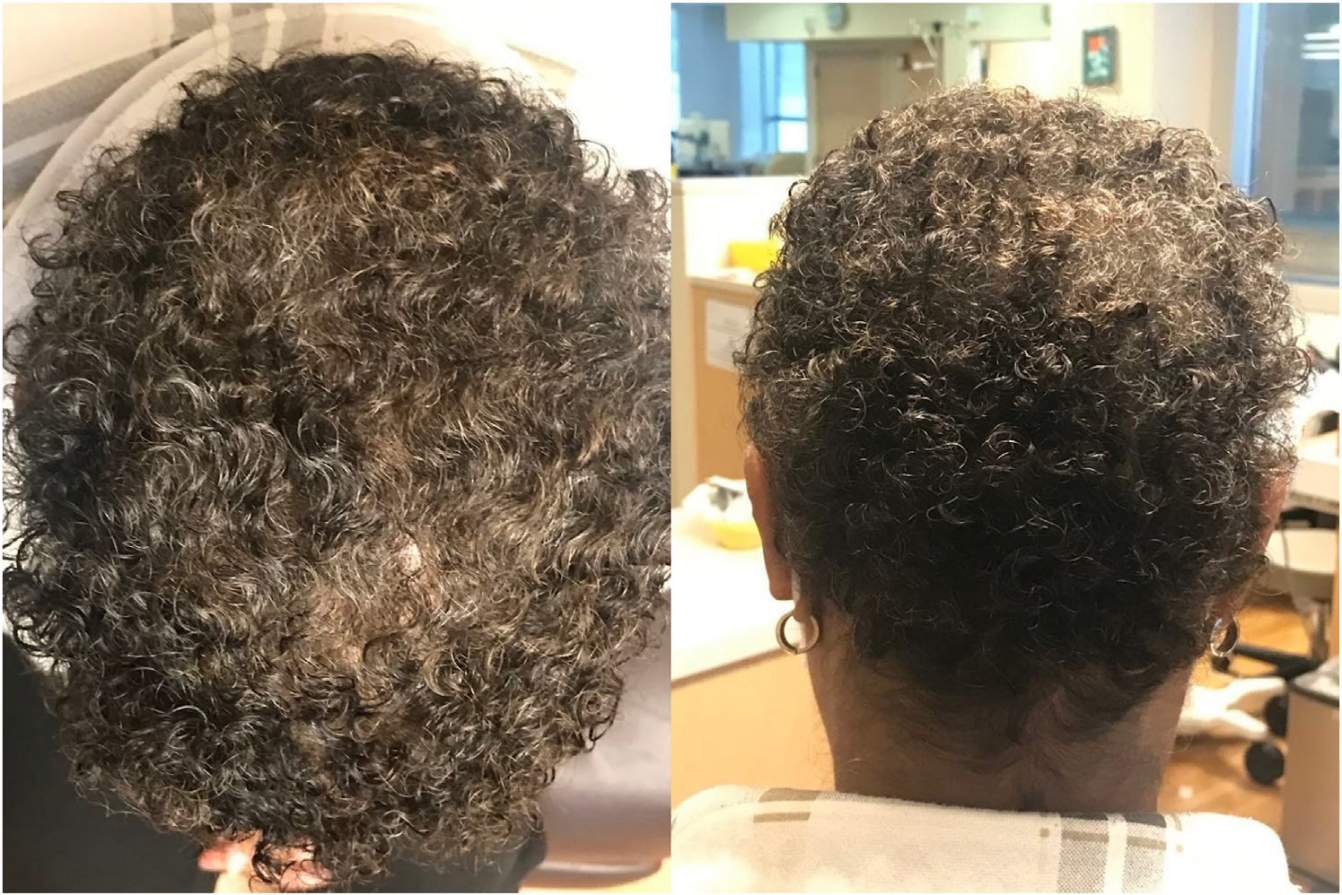
Grade 0 alopecia (no hair loss) after cycle 2 of taxotere + cyclophosphamide regimen.

**Fig 3:**
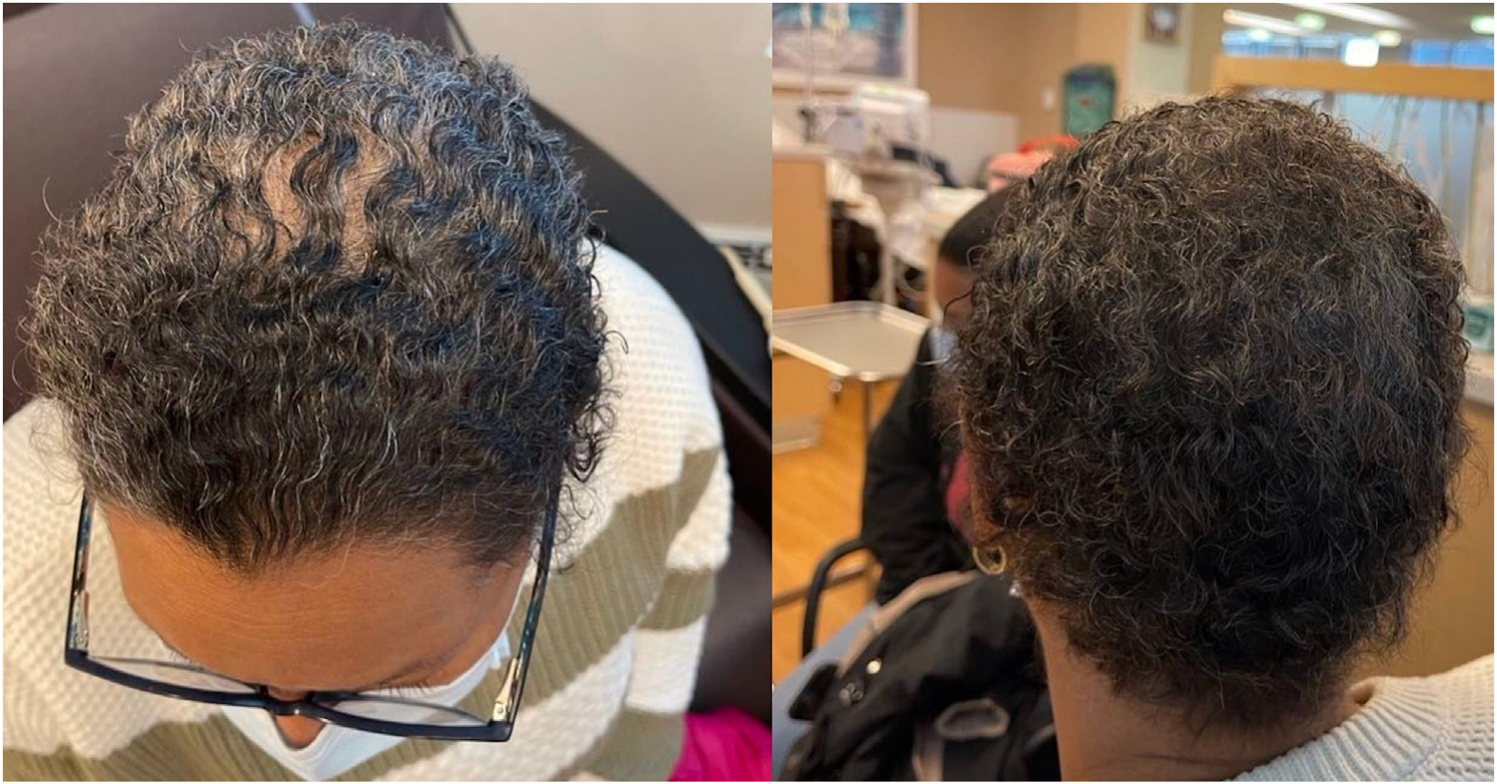
Grade 1 alopecia (minimal hair loss) after cycle 3 of taxotere + cyclophosphamide regimen.

**Fig 4:**
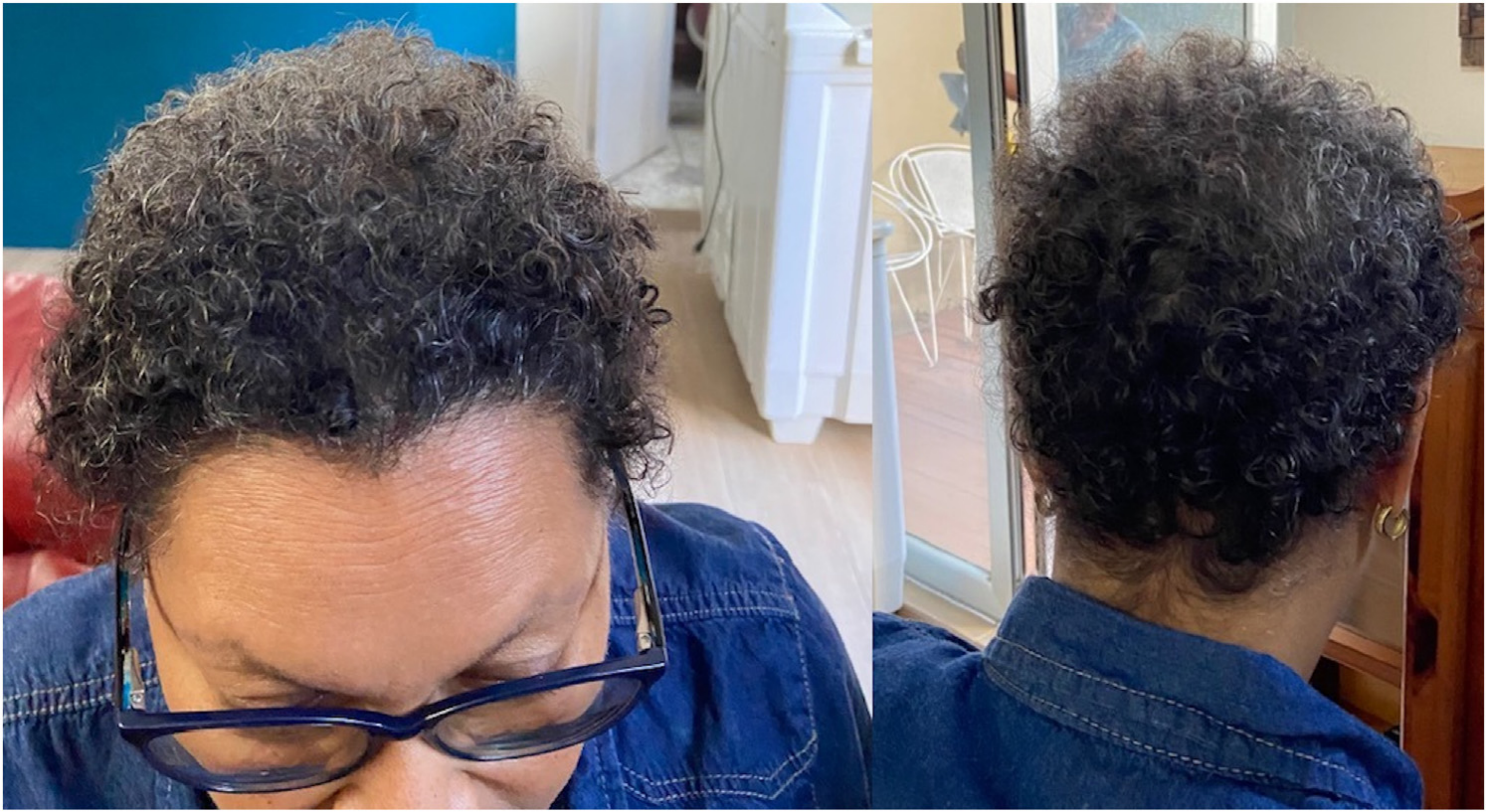
Grade 1 alopecia (minimal hair loss) after cycle 4 of taxotere + cyclophosphamide regimen.

The Paxman cooling system was utilized. The CC and cover were placed on the patient’s head and the chinstrap was secured. The CC was then connected to the computerized cooling unit that maintains the CC at a stable temperature between −1 and −4° C during all 3 stages of the SC process. Precooling was initiated 45 minutes prior to the infusion. The patient received all her premedication (dexamethasone, diphenhydramine, and omeprazole) concurrently with precooling. Cooling was continued for the entire chemotherapy infusion and for 90 minutes after the infusion. The CC was left in place for 5 minutes to allow for warming of both the scalp and the CC to aid in removal. The patient had grade 0 alopecia (no hair loss) at baseline (Fig 1) and after completion of her first 2 cycles of chemotherapy (Fig 2). After her third cycle, she experienced grade 1 alopecia with perceptible thinning of the midscalp, which was not obvious from a distance but only upon close inspection of the scalp. (Fig 3). Her alopecia remained stable upon completion of her fourth cycle of chemotherapy (Fig 4)

## Discussion

Prior studies have found SC to be highly effective in preventing CIA. However, minority representation was limited. A recent study found that SC was less efficacious in patients with skin of color, likely because patients with skin of color have predominantly types 3 (curly) and 4 (kinky) hair, which tend to become bulkier when wet and can interfere with SC cap fitting.^7^ Although types 1 (straight), 2 (wavy), 3 (curly), and 4 (kinky) hair have identical chemical properties, they have different physical properties, such as shape, texture, and density.^8^ Although these differences in hair texture and shape may appear trivial, they may significantly impact SC success and warrant different SC hair preparation techniques.

CCs need to be in close contact with the scalp to allow for maximum heat exchange. Gaps between the scalp and CC create air pockets of insulation, trapping heat, and impairing heat exchange. Bulkier hair prevents the CC from being flushed with the scalp because it creates a physical barrier that keeps the scalp and the CC from being in close contact.^8^ To combat this bulking effect of types 3 and 4 hair, we utilized hair styling techniques that aimed at minimizing hair volume. Although conditioner was previously only applied to the outer surface of the hair to prevent the CC from sticking to the hair during CC removal, we incorporated a conditioner-water emulsion throughout this patient's hair, using the conditioner to act as a means of minimizing hair volume to increase CC to scalp contact. Hair twisting acted as another means of minimizing hair volume as well.

The patient reported here had short hair, and more research is needed to test this technique in people with a variety of hair types and lengths. Improving SC efficacy in Black women has the potential to increase the number of women choosing to undergo lifesaving chemotherapeutic treatment and reduce existing disparities in cancer care. We encourage centers offering SC to recognize that hair preparation should not be a one-size-fits-all approach and individual adjustments may be needed depending on each patient's hair.

## Conflicts of interest

None disclosed.
